# Erratum

**DOI:** 10.1111/jcmm.12203

**Published:** 2013-12-25

**Authors:** M Pini Collino

In 1, all the authors’ names were presented in reverse order. The names should read, in last name, first name format, as follows:

Collino, Massimo

Pini, Alessandro

Mastroianni, Rosanna

Benetti, Elisa

Lanzi, Cecilia

Bani, Daniele

Manoni, Marco

Fantozzi, Roberto

Masini, Emanuela

We wish to apologise for any misunderstanding or inconvenience caused.

## References

[b1] Collino M, Pini A, Mastroianni R (2012). The non-anticoagulant heparin-like K5 polysaccharide derivative K5-N, OSepi attenuates myocardial ischaemia/reperfusion injury. J Cell Mol Med.

